# Nanoscale Characterization of Graphene Oxide-Based Epoxy Nanocomposite Using Inverted Scanning Microwave Microscopy

**DOI:** 10.3390/s22249608

**Published:** 2022-12-08

**Authors:** C. H. Joseph, Francesca Luzi, S. N. Afifa Azman, Pietro Forcellese, Eleonora Pavoni, Gianluca Fabi, Davide Mencarelli, Serena Gentili, Luca Pierantoni, Antonio Morini, Michela Simoncini, Tiziano Bellezze, Valeria Corinaldesi, Marco Farina

**Affiliations:** 1 Department of Information Engineering, Università Politecnica delle Marche, Via Brecce Bianche, 60131 Ancona, Italy; 2 Department of Materials, Environmental Sciences and Urban Planning, Università Politecnica delle Marche, Via Brecce Bianche, 60131 Ancona, Italy; 3 Department of Industrial Engineering and Mathematical Science, Università Politecnica delle Marche, Via Brecce Bianche, 60131 Ancona, Italy

**Keywords:** inverted scanning microwave microscopy (iSMM), nanoscale electrical properties, graphene oxide (GO), epoxy nanocomposites

## Abstract

Scanning microwave microscopy (SMM) is a novel metrological tool that advances the quantitative, nanometric, high-frequency, electrical characterization of a broad range of materials of technological importance. In this work, we report an inverted near-field scanning microwave microscopy (iSMM) investigation of a graphene oxide-based epoxy nanocomposite material at a nanoscopic level. The high-resolution spatial mapping of local conductance provides a quantitative analysis of the sample’s electrical properties. In particular, the electrical conductivity in the order of ∼$10^{-1}$ S/m as well as the mapping of the dielectric constant with a value of ∼4.7 ± 0.2 are reported and validated by the full-wave electromagnetic modeling of the tip–sample interaction.

## 1. Introduction

The scanning microwave microscope (SMM) [1] is one of the scanning probe microscopy (SPM) techniques [2], a family that includes the well-known atomic force (AFM) and scanning tunnelling (STM) microscopes. In fact, SMM makes use of other SPM techniques to control and keep constant the distance between the probe and the sample. Thus, in SMM, the probe has a twofold use: it scans the surface of the sample to map the topography, and it acts as an antenna for the microwave signal. A vector network analyser (VNA) generates (incident signal) and collects (reflected signal) the microwaves that have been interacting with the sample. As a matter of fact, the SMM technique evaluates the probe–sample interactions using an evanescent field, which decays exponentially from the probe (source) [2]; a sharp tip can be used to achieve the best resolution since the electromagnetic field is almost singular near a thin edge.

Imtiaz and Anlage [3] combined a standard tunnelling microscope with a microwave signal: the feedback circuit of the STM was used to keep the probe at a given distance from the sample, and the microwaves were collected in a reflectometric setup that allowed for a nanometric resolution. Other authors used AFM with a conductive tip to control the feedback circuits and to introduce microwaves [4], while other researchers presented a “scanning impedance microscope”, exploiting a VNA and an atomic force controller [5,6,7]. The devices take advantage of the very large dynamics of the VNA and make possible accurate quantitative measurements of the complex impedance [8]. Generally, the above-described systems are intrinsically narrow-band, i.e., a resonator (or an interferometer) is introduced to increase sensitivity in a limited frequency range; on the contrary, a system can perform local microwave spectroscopy in an extended frequency range and in time-domain analyses. Our group has been a pioneer in SMM for several years [9,10,11,12,13,14,15,16,17,18]. In our first implementation, a platinum tip was used to trace the tunnelling current and to record the microwave signal; the tunnelling circuitry was used to keep a tip/sample distance of a few nanometres. Meanwhile, the tip also acts as a microwave antenna. In this way, due to the presence of the sample, it was possible to sense local changes in the impedance by measuring the reflection coefficient ($S_{11}$). Moreover, thanks to the multiple frequency approach, the sensitivity of the whole system was improved by exploiting the correlation of the images obtained at several close frequencies and by using the time-domain reflectometry [11,14]. This methodology can be comprehended in terms of “spread-spectrum”: by an inverse Fourier transform, the information that is spread inside a frequency spectrum is recovered by collapsing it at a single time instant. STM-assisted SMM provided very high-quality images, even though this apparatus was extremely challenging when dealing with poorly conducting samples, such as biological ones, and even more difficult when used in liquid [19].

More recently, our group developed a new setup called the inverted SMM (iSMM) [12], which is able to overcome some limitations of the original SMM. The structure of the inverted SMM is low-cost, easily available, and compatible with the liquid environment and with poorly conducting samples. iSMM has substantial advantages over the standard SMM, such as higher sensitivity and wider dynamic range, and the latter can be achieved by exploiting two port measurements. In the inverted SMM, as the name suggests, there is an inversion of the probe and the sample holder; the latter is a transmission line over which microwave signals are measured, and the SPM probe perturbs the line while sensing the sample. In detail, the conductive scanning probe (AFM or STM) is always AC-grounded, and the microwave signal is injected through a transmission line where the sample is placed (sample holder). Both the input and output of the transmission line are connected to a VNA, such that the reflected and transmitted signals ($S_{11}$ and $S_{21}$, respectively) can be measured [12,15,16]. It is important to underline that general iSMM is broadband, enabling frequency spectroscopy, time domain analyses, and microwave tomography. So far, the SMM has been used to characterize living biological cells despite the challenges operating in physiological buffers [20]. Furthermore, it has also been used on subcellular organelles such as mitochondria [9], which are responsible for cell respiration and energy production. The iSMM has proved to be able to overcome the limitations of in-liquid operation by demonstrating the first successful microwave imaging of live cells [12] or subcellular organelles [18] in a physiological buffer [12].

The electrical and electronic applications of graphene-based composites have been gaining attention for their wider range of applications in industries [21]. These composite materials are typically made by mixing an electrical insulating material, such as epoxy, with electrically conductive graphene or graphene oxide (GO) as filler material. The higher filler content results in an increase in electrical conductivity [22,23]. In this work, the high-frequency electrical properties of epoxy and a GO-based composite material are studied at the nanoscopic level.

## 2. Materials and Methods

### 2.1. Experimental Setup

**Figure 1:**
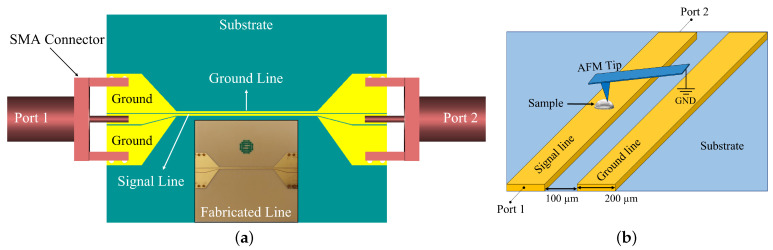
The experimental setup of the inverted SMM: (**a**) The slot-line design (inset shows the fabricated slot line). (**b**) Schematic description of the grounded metallic AFM tip and the slot line as a sample holder.

The iSMM setup is basically composed of an AFM (NT-MDT Solver Pro-47) and a planar transmission line, which also serves as a sample holder. Figure 1a shows the slot line utilised in our setup interfaced with the Vector Network Analyser (VNA) through SubMiature version A (SMA) flange connectors and coaxial cables. The VNA acts as a source and detector system for the microwave signal, and it records both the reflection ($S_{11}$) and transmission ($S_{21}$) coefficients. The AFM probe is a gold-coated NSG03/Au tip from TipsNano, with a tip apex radius of 35 nm and operated in semi-contact mode. The AFM probe is grounded and locally induces perturbation in the line impedance along the signal line of the transmission line during the AFM scan, as shown in Figure 1b. The sample is deposited onto the signal line. Taking advantage of the interfacing of AFM with VNA measurements, iSMM can simultaneously provide the topography as well as the microwave imaging (Amplitude and Phase) with the spatial resolution of an AFM.

The slot line is deposited onto a 30 mm × 30 mm low-loss laminate substrate with a thickness of 512 µm. The metallic part is composed of a layer of copper (30 µm) and nickel (5 µm), with a 30 nm gold coating on top. The top part of the line is made to have a smooth surface with a very low roughness, which is a requirement for the scanning of the nanoparticles with AFM. The signal line has a width of 200 µm, with a gap of 100 µm to the ground line. The slot line is designed to have resonances by introducing a transition, from the typical system impedance of 50Ω to a higher characteristic impedance (∼83 Ω). The introduced mismatch produced by increasing the characteristic impedance of the slot line is expected to enhance the sensitivity of the iSMM measurements at the resonance frequencies. The measurement sensitivity also depends on the position of the sample along the line as a result of the maxima of the electric field with respect to the frequency of operation.

**Figure 2:**
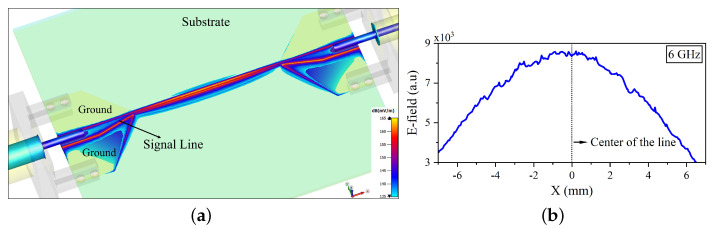
Simulated 3D electric field map. (**a**) The electric field map of the slot line at 6GHz. (**b**) The e-field profile taken along the length of the signal line.

A 3D model of the slot line is simulated with a commercial finite-difference time-domain (FDTD)-based electromagnetic solver CST Microwave studio. The resulting electric field map at 6 GHz is shown in Figure 2a. The field profile taken along the length (X direction) of the signal line and centred in the Y direction is displayed in Figure 2b. The field reaches its maximum at the centre of the line and is uniform up to 4 mm along the line. This ensures that the sample is deposited onto an area of the slot line where the electric field is at its maximum within this frequency range.

### 2.2. Sensitivity of the iSMM System

The sensitivity of the system for the optimal working frequency range for an iSMM scan can first be identified by predicting the signal-to-noise ratio (SNR), as defined in Equation (1) expressed in the dB scale. Here, ($S_{ij}$) is the complex value of the reflection ($S_{11}$) or transmission ($S_{21}$) coefficient, which includes the amplitude and phase details measured at two distinct tip–sample positions: (i) initial tip–sample position after the completion of the AFM approach operation for the semi-contact mode ($d_{1}\approx 30$ nm), and (ii) lifted up in the air ($d_{2}\approx 3$ µm) by switching off the feedback system. Each measurement has to be repeated 500 times and averaged in order to minimize the noise, with σ as the standard deviation.

(1) $$\begin{array}{c} SNR\left(S_{ij}\right)=20log\left|\frac{S_{ij}\left(d_{1}\right)-S_{ij}\left(d_{2}\right)}{\sigma \left[S_{ij}\left(d_{1}\right)\right]}\right| \end{array}$$

**Figure 3:**
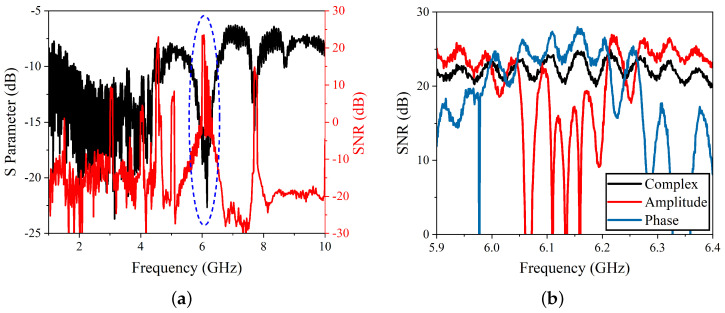
Signal-to-noise ratio of the iSMM microscopy system. (**a**) The $S_{11}$ and complex SNR in the frequency range of 1–10 GHz. The SNR peaks around the resonance frequency close to 6 GHz. (**b**) The SNR complex, amplitude, and phase in the chosen frequency band for the iSMM scan.

Typically, good quality iSMM images can be obtained with an SNR over 20 dB. Figure 3a shows the measured $S_{11}$ and the complex SNR in the range of 1–10 GHz. The sensitivity peaks around the resonance frequencies, and SNR reaches more than 20 dB close to 6 GHz in the circled area of Figure 3a. As good sensitivity in a frequency band was spotted, the measurement was narrowed down to the 5.9 GHz–6.4 GHz band. The measured complex, amplitude, and phase of the SNR is shown in Figure 3b. Since the SNR is higher in this frequency band, it was selected for the iSMM scanning operation.

### 2.3. Sample and Microstructure Analysis

The sample material is a two-component epoxy system. The epoxy resin was supplied by Sicomin and is composed of an SR 1660-type resin and an SD 2630-type catalyst; it is characterized by good chemical resistance, oxidation resistance, and a low exothermic peak reinforced with a hardener dispersed into the matrix. This type of resin is commonly used in the manufacturing of parts or tools that operate under high-temperature environments of up to 160 °C. Graphene Oxide (GO) in the form of flakes was supplied by Hygraner.

An epoxy-based carbon composite system containing 3.0 wt % of GO was prepared by adding GO to the epoxy resin and dispersing under mechanical stirring. The concentrations of the GO were calculated from the total weight of the reactive system (epoxy resin + hardener). The nano-filled epoxy samples underwent an oven-curing process by applying the following thermal cycle [24]: 48 h at room temperature, 16 h at 60 °C, and 6 h at 100 °C.

The fractured surfaces of the manufactured Epoxy/GO were analysed by FESEM (Supra 40-Zeiss, Oberkochen, Germany) after gold sputtering and with an accelerating voltage of 5 kV. The fractured surfaces of the Epoxy and the Epoxy/3%GO were gold-coated with an Agar automatic sputter coater and then analysed.

**Figure 4:**
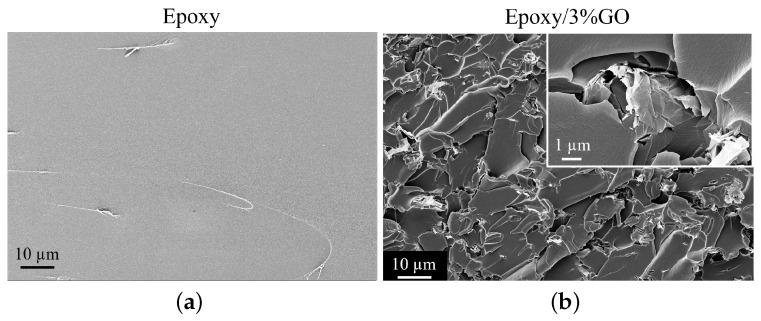
FESEM images of the fractured cross-sections of the (**a**) Epoxy and (**b**) Epoxy/3 wt%GO systems.

The compactness of the epoxy systems was investigated by observing the internal surfaces. The inter-facial status of the epoxy and the fillers play a key role in the overall performance of the nanocomposite systems [25]. The effect of the nanocomposite preparation method on dispersion quality and on the different content of the GO sheets in the epoxy-based samples is investigated by FESEM. Figure 4 shows cross-section images of pure epoxy and epoxy/3.0%GO. The epoxy resin (Figure 4a) has a uniform and smooth fractured surface without defects, suggesting a brittle fracture and low-fracture toughness [26]. The epoxy with 3.0 wt % of GO is characterized by the typical graphene oxide aggregates (Figure 4b and inset) [25]. The sample was then converted into a fine powder form in order to be able to deposit it onto the sample holder of the iSMM signal line.

## 3. Results and Discussion

### 3.1. iSMM Imaging

**Figure 5:**
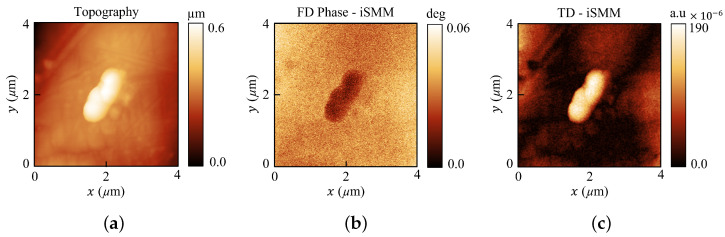
iSMM images of a nanocomposite particle. (**a**) AFM topography. (**b**) Frequency domain phase signal image of iSMM recorded at a frequency of 5.99 GHz. (**c**) iSMM time domain image.

The iSMM scan was performed in the frequency band of 5.9 to 6.4 GHz, and it recorded the reflected microwave signal ($S_{11}$). Simultaneous AFM topography and iSMM frequency domain (amplitude and phase) images were acquired by scanning an area of 4×4 µm, with a pixel size of 256×256 as shown in Figure 5. Each scan was recorded with 512 frequency points in the working frequency band. Figure 5a shows the tilt-corrected AFM topography image of a nanocomposite particle. Figure 5b shows the iSMM phase image (filtered) obtained at 5.99 GHz.

As mentioned in the introduction, one of the important advantages of both our standard and the iSMM system is their ability to acquire time domain images by applying an inverse Fourier transform to the obtained complex frequency-domain data. The time evolution of the electromagnetic field allows us to gate out all the unwanted signals and obtain only the tip–sample interaction [14]. In addition to that, the time-domain image combines details of the whole frequency band. This time-domain approach enhances the image quality in a remarkable way and reveals minute details about the surface and sub-surface of the sample, which cannot be seen with the use of simple AFM or STM topography [11]. Figure 5c shows the time domain image, which displays impressive quality compared to the AFM topography or frequency domain images.

### 3.2. iSMM Calibration

An important aspect of iSMM in getting quantitative electrical information/properties of the sample from the obtained frequency domain images is the accurate calibration of the tip–sample system in order to evaluate the local impedance or admittance measurement at the nanometric scale. Calibrating microwave measurements involving VNA usually requires a set of well-known loads that permit us to identify and eliminate the error network, which contains all the unwanted interactions other than a desired local tip–sample interaction.

As the microscopy system involves measurements at the microscopic level, one cannot simply use the so-called standard calibration tools to calibrate the microwave microscopy measurements. To this end, a specific calibration procedure was implemented without the use of any calibration sample through the measurement of the capacitance by moving/approaching the tip above a piece of metal and comparing it with an analytical model of a sphere on top of a metallic ground plane [13]. This method was also extended to the iSMM setup to calibrate the obtained images [16]. By using this calibration approach, one could acquire a calibrated local tip–sample admittance.

**Figure 6:**
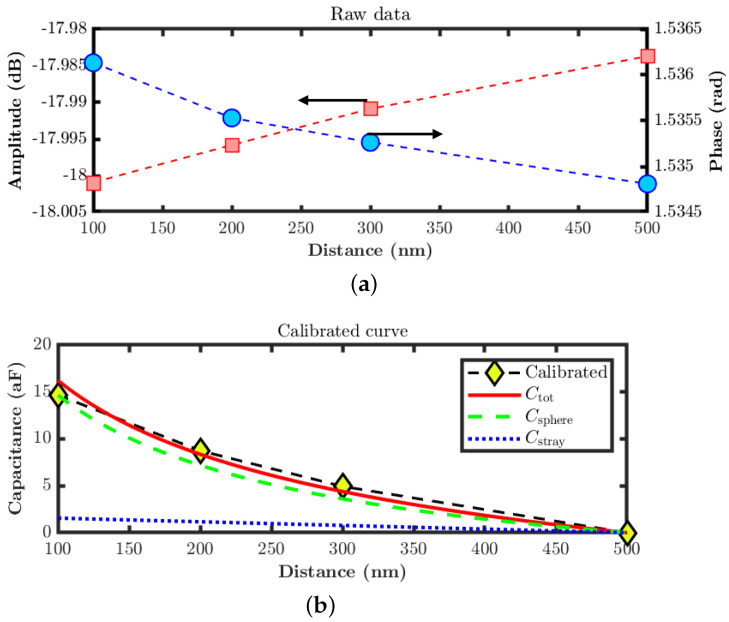
Approach curve calibration. (**a**) Raw data of the $S_{11}$ approach curve on top of the signal line, measured at a frequency of 5.99 GHz. (**b**) Calibrated capacitance curve compared with an analytical model of the tip as a sphere.

The calibrated approach curves are presented in Figure 6. Figure 6a shows the amplitude and phase signals of the $S_{11}$ approach curve performed at different tip–sample distances on the same scan, which correspond to the tip position of the signal line over a metallic surface at a frequency of 5.99 GHz. Figure 6b shows the calibrated capacitance curve acquired from the approach curve data, which agrees well with the tip–sample analytical model, including a stray contribution coming from other parts of the tip. The estimated effective tip radius for calibrating the curve is 300 nm, and the stray capacitance value used to fit the curve is $3.9\times 10^{-12}$ F/m. This calibration curve was used to calibrate the following $S_{11}$ images obtained during the same operation.

**Figure 7:**
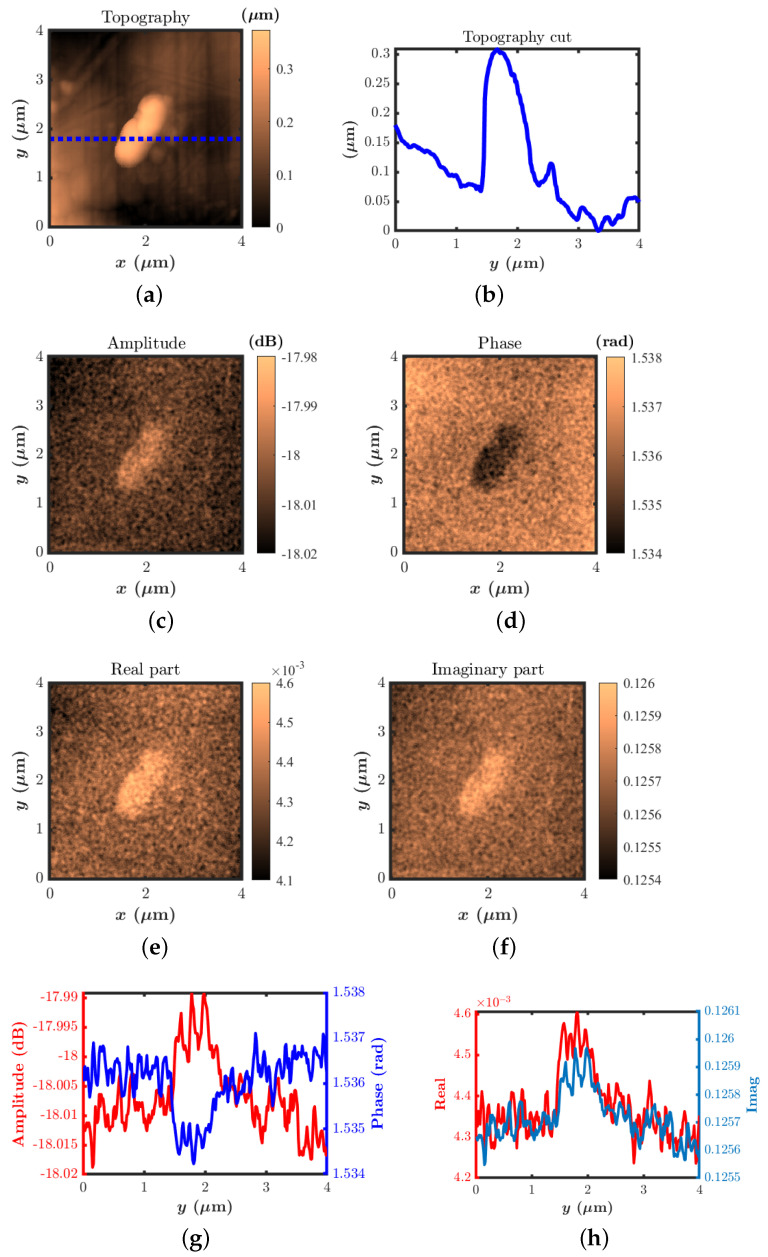
(**a**) AFM topography. (**b**) Line profile of the AFM topography along the dotted line shown in the topography image. Frequency-domain images of $S_{11}$ obtained at 5.99 GHz. (**c**) Amplitude, (**d**) phase, and (**e**) real part of $S_{11}$; (**f**) imaginary part of $S_{11}$. Corresponding line profiles along the same cross-section as that in the topography (**g**,**h**).

Figure 7 shows the simultaneously acquired AFM topography and raw data of the reflection $S_{11}$ measurement without the application of any filtering other than tilt correction to the images, as one of the requirements for the calibration is to have unaltered data. Figure 7a,b show the AFM topography and the line profile along the centre of the image, indicating the nanocomposite particle positioned between 1.5 and 2.5 µm, with a height of around 300 nm. The $S_{11}$ images acquired at a frequency of 5.99 GHz, where the sensitivity was high enough for the acquisition of quality images, show good contrast, as can be seen in Figure 7c–h.

**Figure 8:**
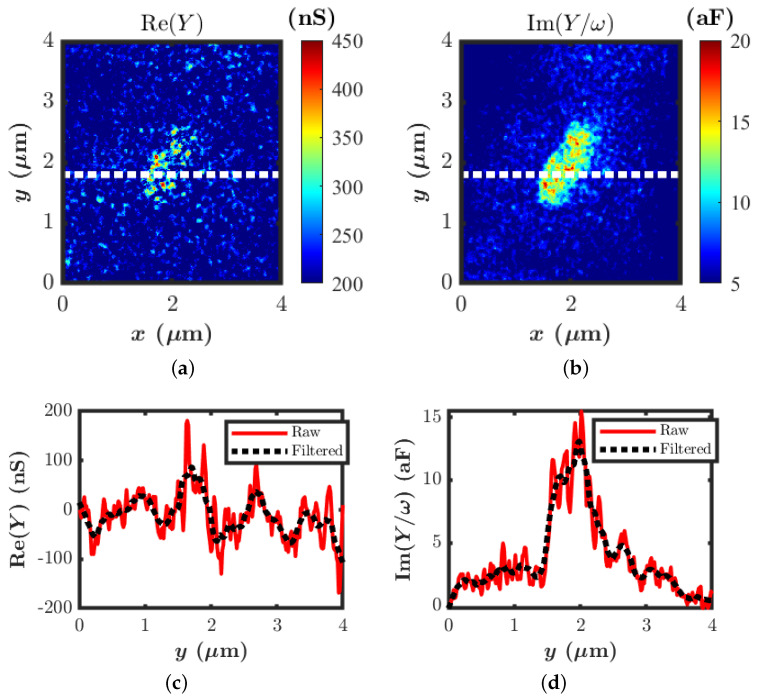
Calibrated local admittance. (**a**) Real part of the admittance. (**b**) Imaginary part of the admittance. (**c**) Line profile corresponding to the Re (Y). (**d**) Line profile corresponding to the Im (Y)/ω.

The calibrated real and imaginary parts of the local admittance are shown in Figure 8a,b. Figure 8c,d illustrate the corresponding line profiles along the x-direction. The Re(Y) has a peak value of around 180 nS on the line profile compared to the bare signal line surface, where it reaches zero when the tip sees the metal substrate separated only by air. On the other hand, the Im (Y)/ω shows the peak value of around 15.4 aF on the line profile. To extract the electrical conductivity, a 2D axis-symmetric, full-wave finite element methodology (FEM)-based electromagnetic simulation of a tip–sample system was performed using the commercial software COMSOL multiphysics in order to directly evaluate the tip–sample admittance as a function of the sample dielectric constant and conductivity.

**Figure 9:**
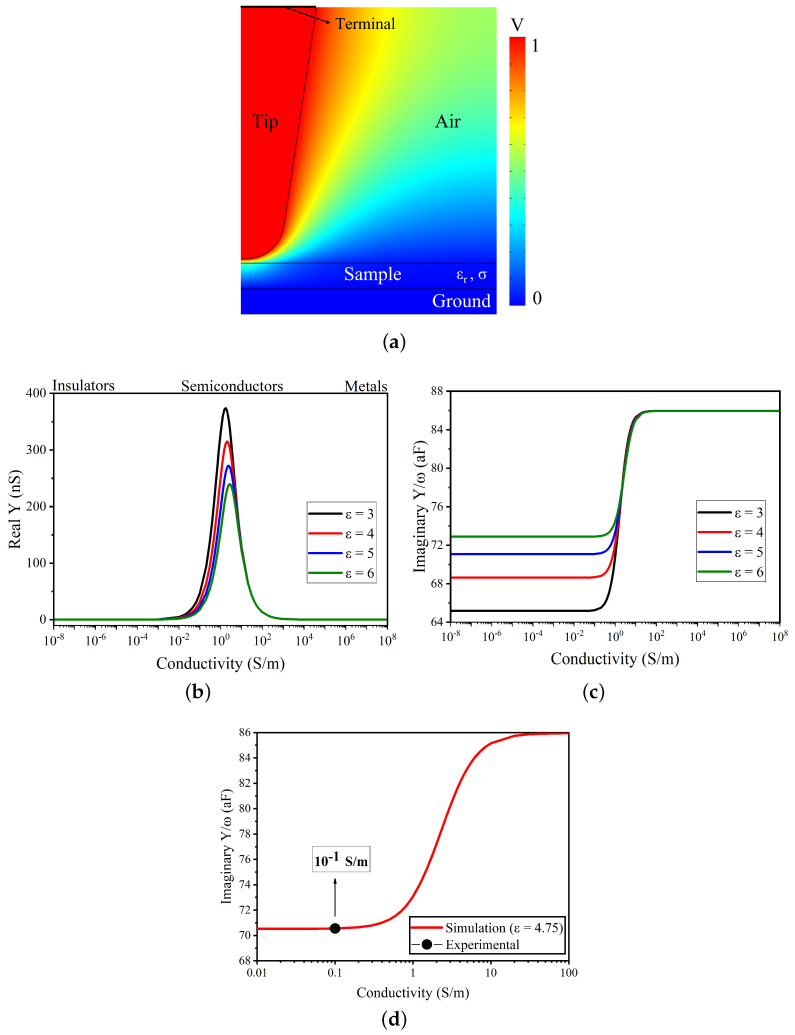
A 2D axis-symmetric model of the tip–sample system. (**a**) Potential field distribution and the tip–sample model. (**b**) Simulated response of Re (Y) for different $\epsilon_{r}$ values. (**c**) Imaginary part of Im(Y)/ω. (**d**) Simulated imaginary part of the Im(Y)/ω of the tip–sample admittance at 5.99 GHz corresponding to an $\epsilon_{r}$ value of 4.75 and compared with the calibrated experimental value of Im(Y)/ω.

The model considers all the parameters similar to the experimental values to have a tip radius of 300 nm, a tip–sample separation of 30 nm, and a sample thickness of 300 nm. Figure 9a shows the modelled 2D axis-symmetric simulation setup and the field distribution. Figure 9b,c show the simulated values of Re(Y) and Im(Y)/ω for different combinations of $\epsilon_{r}$ and σ, which demonstrates the sensitivity of the microscope model to the local variations of the sample’s electrical properties. Figure 9d shows the comparison of the experimental point with the simulated curve. The peak value obtained from the cross-section profile of the Im(Y)/ω data is around 15.4 aF; as the sides of the sample is a metallic signal line, this value has to be subtracted from the maximum value of the simulated curve corresponding to the metal. In doing this, the value of 70.55 aF was obtained: this corresponds to a value of around 0.1 S/m in the Im(Y)/ω curve and to an $\epsilon_{r}$ value of 4.75. The obtained conductivity value is similar to the DC conductivity value reported in [23] for the graphene oxide based epoxy.

The local dielectric constant of the sample can be derived from the calibrated Im (Y)/ω image by carefully applying a proper correction to the topography crosstalk contribution. The dielectric constant for a tip in contact with the sample can be evaluated by subtracting the topography crosstalk contribution $C_{cross}$ from the calibrated Im(Y)/ω using the following formula [27]:

(2) $$\begin{array}{c} \epsilon_{contact}=e^{\frac{ImY/\omega -C_{cross}}{2\pi \epsilon_{0}R}} \end{array}$$

Here, $C_{cross}$ is defined as the capacitance between the tip and the metal ground when their distance is relative to the sample height (h), and R is the radius of the tip. Since our iSMM measurements were performed in the semi-contact mode with a tip–sample distance (z) of 30 nm, the differential capacitance equation related to the dielectric constant corresponding to the non-contact mode of operation given in the literature [27] was modified and obtained the following simple expression:

(3) $$\begin{array}{c} \epsilon_{non-contact}=\frac{\epsilon_{contact}}{1+\frac{z}{h}\times \left(1-\epsilon_{contact}\right)} \end{array}$$

**Figure 10:**
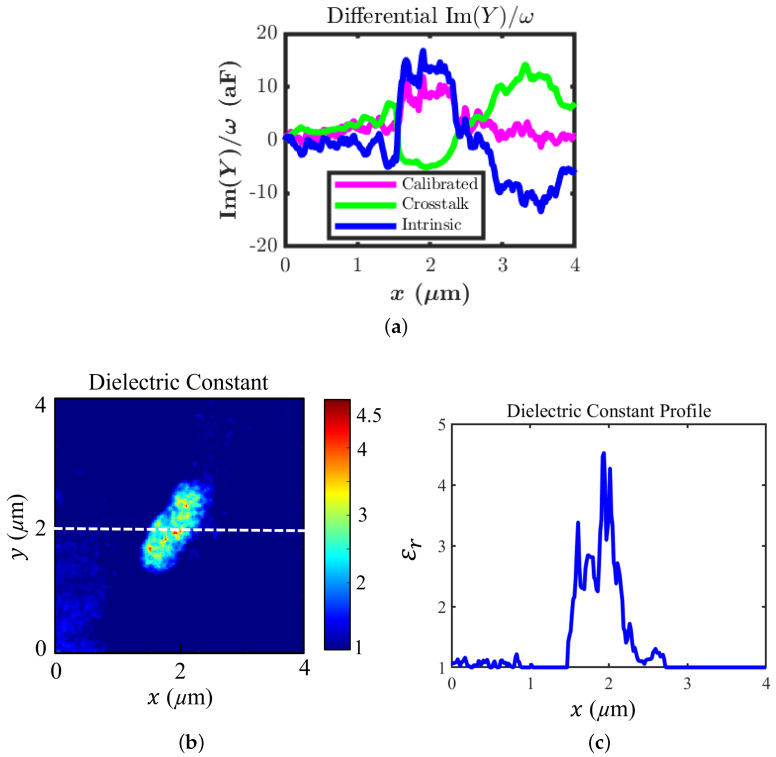
(**a**) Line profiles of the calibrated Im(Y)/ω (purple), crosstalk contribution coming from other parts of the tip (green), and intrinsic Im(Y)/ω (blue). (**b**) The extracted dielectric constant map for the non-contact mode. (**c**) The line profile of the dielectric constant along the centre of the scan.

Figure 10a shows the line profiles of the calibrated Im(Y)/ω and the intrinsic Im(Y)/ω along with the crosstalk. The intrinsic Im(Y)/ω was obtained by calculating the difference between the calibrated Im(Y)/ω and the total crosstalk contribution.

The obtained dielectric constant map of the non-contact case using Equation (3) is shown in Figure 10b. The cross-section profile taken from the centre of the dielectric constant map is shown in Figure 10c. The dielectric constant value peaks around the range of 4.5–4.7; outside of the sample, it reaches 1, where the tip only senses the metal signal line separated by air. The experimental dielectric constant value is in good agreement with the value from the electromagnetic simulation results.

## 4. Conclusions

In this work, an investigation of the iSMM system for the quantitative mapping of local electrical properties of a graphene oxide/epoxy nanocomposite was conducted by measuring the local admittance at a high spatial resolution. The electrical conductivity and dielectric constant of the composite material were found to be at ∼$10^{-1}$ S/m and ∼4.7 ± 0.2, respectively. The measured quantitative values were validated by electromagnetic simulations. This demonstrates the ability of the iSMM system to perform a nanoscale, quantitative, electrical characterization of nanocomposite materials.

## Data Availability

Not applicable.
